# The Role of Ferroptosis-Related Molecules and Significance of Ferroptosis Score in Cervical Cancer

**DOI:** 10.1155/2022/7835698

**Published:** 2022-10-30

**Authors:** Pengxiang Li, Xuefeng Lv, Lu Liu, Mengle Peng, Dongchun Qin

**Affiliations:** ^1^Department of Clinical Laboratory, Key Laboratory of Laboratory Medicine of Zhengzhou Province The First Affiliated Hospital of Zhengzhou University, Zhengzhou 450052, Henan, China; ^2^Department of Clinical Laboratory, People's Hospital of Henan University of Chinese Medicine, Zhengzhou 450003, Henan, China; ^3^Department of Clinical Laboratory, Henan No. 3 Provincial People's Hospital, Zhengzhou 450007, Henan, China

## Abstract

**Background:**

Ferroptosis, a form of cell death driven by iron-dependent lipid peroxidation, may be a potential treatment for many cancers, including cervical cancer (CC). However, the regulation of long noncoding RNAs (lncRNAs) in the process of ferroptosis and whether ferroptosis inducers could increase the cytotoxicity of conventional chemotherapy drugs remain to be further elucidated.

**Methods:**

We analyzed the variation of 55 differentially ferroptosis-related genes (FRGs) and the influence of mutations in CC patients. The patients with CC were classified into two ferroptosis clusters by the non-negative matrix factorization (NMF) algorithm. The principal components analysis (PCA) was used to measure the ferroptosis score (FerroScore) in patients with CC. Besides, FerroScore was used to predict the sensitivity of chemotherapy and responses to immunotherapy in patients with CC. Finally, experiments were performed to verify the regulatory effect of AC026790.1 on erastin-induced ferroptosis, as well as the effect of erastin in combination with cisplatin on the toxicity of CC cells (SiHa, HeLa).

**Result:**

There were significant differences in the overall survival and immune cell infiltration between the two ferroptosis clusters. Patients with low FerroScore were more sensitive to chemotherapy drugs such as cisplatin and docetaxel. The low-FerroScore group had higher CD8+ T cell infiltration and immune checkpoint expression, demonstrating that patients with lower FerroScores were more sensitive to immunotherapy, which was consistent with the result of the submap method. In vitro, overexpression of AC026790.1 could promote erastin-induced ferroptosis, and the combination of erastin and cisplatin could increase the toxicity of CC cells.

**Conclusion:**

FerroScore has a potential prognostic value for CC that may provide a reference for chemotherapy and immunotherapy. LncRNA AC026790.1 can influence ferroptosis, and the combination of ferroptosis inducers and chemotherapy drugs can provide a new perspective on cancer treatment.

## 1. Introduction

Cervical cancer (CC) is the primary cause of mortality in the global population of women, with 604,127 new reported patients and 275,000 deaths worldwide each year [1]. Globally, CC ranks as the third most frequent cancer, and it is also the sixth most frequent cancer among women in developed countries, as well as the second most frequent cancer in low-income countries [2]. One of the most important causes of CC is persistent human papillomavirus (HPV) infection, especially oncogenic subtypes HPV 16 and 18 [3]. Although the HPV vaccine is effective in preventing CC, many women in low-income countries remain unprotected due to the significant financial burden imposed by CC screening and vaccination programs [4]. Moreover, patients with recurrent or advanced stages are resistant to radiotherapy and chemotherapy and have a poor prognosis [5]. Therefore, it is crucial to explore additional diagnostic biomarkers and possible therapeutic targets while improving survival.

So far, several types of cell death have been described in previous studies, including autophagy, apoptosis, ferroptosis, necroptosis, and pyroptosis. Thereinto, in ferroptosis, the morphology differs from other cell death patterns and is mainly characterized by distinct mitochondrial shrinkage, increased membrane density, and reduced or disappeared mitochondrial cristae. In 2012, ferroptosis was first proposed by Dr. Stockwell, as a novel form of cell death, and it was caused by an iron-dependent accumulation of large amounts of lethal reactive oxygen species (ROS) [6]. Abundant ROS is produced as a by-product of oxidative phosphorylation and may cause oxidative damage to DNA, membranes, and mitochondrial proteins, resulting in impairment of mitochondrial function [6]. Long noncoding RNAs (lncRNAs) are noncoding RNAs with more than 200 nucleotides in length. Growing evidence has suggested that lncRNAs are involved in the regulation of ferroptosis. For example, Zhang et al. demonstrated that lncRNA OIP5-AS1 promoted prostate cancer proliferation and suppressed ferroptosis through miR-128-3p/SLC7A11 signaling [7]. Another research indicated that lncRNA PVT1 modulated ferroptosis through the miR-214-mediated expression of TFR1 and TP53 [7, 8]. In addition, a recent report has demonstrated that LINC00336 acted as an endogenous sponge of MIR6852 and regulated cystathionine-*β*-synthase (CBS) expression to suppress ferroptosis in lung cancer [9]. Moreover, a study revealed that the knockdown of AP003555.1 and AC000584.1 could inhibit erastin-induced ferroptosis in colon cancer cells [10]. However, there are limited studies of ferroptosis-related lncRNAs in CC. Therefore, it is essential to explore the regulation of ferroptosis-related lncRNAs in CC cells.

Ferroptosis also plays important role in regulating tumor chemotherapy resistance and tumor immunity. Several studies have indicated the potential use of ferroptosis inducers to trigger ferroptosis for cancer therapy, especially for aggressive tumors that are resistant to conventional therapies [11–13]. For instance, propofol may be a potential adjuvant to enhance the chemotherapy sensitivity of CC cells via activating ferroptosis [14]. In addition, Roh et al. revealed that ferroptosis inducers can also act synergistically with some conventional drugs, such as cisplatin, to inhibit tumor growth in a mouse model of head and neck cancer [15]. In the tumor microenvironment (TME), the role of CD8+ T cells can be enhanced by cancer immunotherapy, in turn, immunotherapy-activated CD8+ T cells release cytokines including interferon *γ* (IFN*γ*) [16]. While, IFN*γ* can impair the elimination of lipid peroxides (LPO) via downregulating the expression of SLC7A11 and SLC3A2, leading to a massive accumulation of LPO in tumor cells resulting in ferroptosis. In addition, LPO-dependent ferroptosis in tumor cells can be facilitated by anti-PD-L1 antibodies [16].

In this study, the variation of FRGs and ferroptosis-related signaling pathways were first analyzed. Then, the Non-negative Matrix Factorization (NFM) algorithm was used to divide CC patients into two clusters, and the survival and TME differences between the two clusters were explored. Subsequently, we established a ferroptosis score (FerroScore) and compared the differences in drug sensitivity, TME, and immunotherapy between high- and low-FerroScore groups. Furthermore, we investigated the regulatory role of ferroptosis-related lncRNAs on erastin-induced ferroptosis. Finally, we further demonstrated that the ferroptosis inducer (erastin) in combination with the conventional drug (cisplatin) could improve the cytotoxicity of CC cells.

## 2. Material and Methods

### 2.1. Data Acquisition and Preparation

RNA-seq data of 309 CC samples, comprising 3 normal and 306 tumor cases, and clinical features were downloaded from the Cancer Genome Atlas (TCGA) database (https://portal.gdc.cancer.gov/) ending in September 2021. Differentially expressed molecules, including lncRNAs and mRNAs between CC and normal cervical tissues, were identified according to the cut-off value of |fold-change (FC)| > 1 and *P* < 0.05. 259 FRGs were identified from the FerrDb (http://www.zhounan.org/ferrdb/) [17], which contained the most detailed list of FRGs. Ferroptosis-related lncRNAs were identified according to the criteria of *P* < 0.001 and Pearson correlation coefficient > 0.3 (|*R*| > 0.3). GSCALite (http://bioinfo.life.hust.edu.cn/GSCA/#/) website was conducted to analyze and visualize some TCGA data.

### 2.2. Non-Negative Matrix Factorization (NMF)

To explore the relationship between the clusters and FRGs in CC, we used the “NMF” package of R software (version 4.0.3) [18] to cluster the CC patients into 2 clusters (Cluster 1 and Cluster 2). The optimal value of clusters was selected based on the cophenetic coefficient.

### 2.3. Evaluation of FerroScore

FerroScore was calculated by the PCA [18] algorithm based on the expression of 55 FRGs in CC patients. The FerroScore formula: FerroScore = ∑(PC1 + PC2). Then, the ability of FerroScore was used to predict tumor immunity, drug resistance, and response to immunotherapy in CC patients.

### 2.4. Pathway Analysis

Gene Ontology (GO) and Kyoto Encyclopedia of Genes and Genomes (KEGG) were analyzed by the R package “ClusterProfiler.” The absolute quantification of some cancer-related signaling pathways was evaluated to compare the differential pathways between high- and low-FerroScore groups by the “GSVA” package.

### 2.5. Drug Sensitivity

The R package “PRRophetic” was used to estimate the drug sensitivity of IC50 for chemotherapeutic agents in CC patients. In addition, chemotherapy drug sensitivity was predicted from the Genomics of Drug Sensitivity in Cancer (GDSC). Data analysis and visualization of FRGs and chemotherapy drug sensitivity were acquired from the GSCALite website.

### 2.6. Estimation of the Immune Features of TME

CIBERSORT [19, 20] is an algorithm that represents the abundance of complex immune cells based on preprocessed gene expression profiles. The ESTIMATE [21] algorithm is used to calculate the scores of immune, stromal, and ESTIMATE. Based on expression data, single sample gene set enrichment analysis (ssGSEA) [22] was used to quantify the level of infiltration of 16 immune cells in each CC patient sample. The Tumor Immune Dysfunction and Exclusion (TIDE) algorithm was used to calculate the TIDE scores in patients with CC which assessed the immune mechanisms of dysfunction of cytotoxic T lymphocytes (CTLs) and rejection of CTLs by immune suppressors. Besides, the TIDE algorithm could predict the immunotherapeutic response in cancer patients. The submap algorithm of GenePattern was used to predict the response to immune checkpoint inhibitors of PD-1 and CTLA4 in high- and low-FerroScore groups.

### 2.7. Collection of CC Tissues and Culture of Cell Lines

20 pairs of CC tissues and their adjacent paracancerous tissues used for the experiment were obtained from the First Affiliated Hospital of Zhengzhou University after surgical resection, from December 2020 to December 2021. The patients were diagnosed according to the 2020 NCCN Guidelines for CC [23] and pathological results and were not treated with radiotherapy or chemotherapy. All the tissues were stored at −80°C. This study was authorized by our hospital ethics committee (Ethics No. 2018-KY-28). And all patients had consented and signed the informed consent.

CC cell lines (Hela and Siha) were purchased from Procell Life Science & Technology Co., Ltd. The cells were cultured in high-glucose Dulbecco's Modified Eagle's Medium (DMEM) with 10% fetal bovine serum (FBS, Vivacell, Shanghai, China, C04001-500). The cell lines were cultured in a CO_2_ incubator at 37°C and 5% CO_2_.

### 2.8. Quantitative Real-Time Polymerase Chain Reaction (qRT–PCR)

Firstly, Trizol (CWBIO, China, CW0580) was applied for total RNA extraction of the tissues and cells. Subsequently, reverse transcription of the total RNA from the previous step was performed, using a reverse transcription kit (Takara, Kyoto, Japan, RR047A), to synthesize cDNA. Finally, qRT–PCR was conducted with the SYBR Green Master Mix (Yeasen, Shanghai, China, 11202ES08) to quantitate the cDNA. GAPDH was an internal reference for calibration. The method of 2^−ΔΔCt^ was chosen to calculate the relative expression of lncRNAs. The primers were shown in Supplementary Table S1.

### 2.9. Cell Counting Kit-8 (CCK-8) Assay

The cells in the exponential growth phase were seeded at 2 × 10^3^ per well into 96-well plates. After 24 hours, the cells were treated with a dose of erastin or cisplatin. After treating the drugs for the appropriate time, the medium was removed from each well and replaced with a fresh medium with a 10% CCK-8 (10 *μ*l) reagent (Dojindo Laboratories, Japan, CK04), and the OD450 was measured after 2 hours of incubation. Finally, the cell viability was calculated from OD450.

### 2.10. Measurement of Lipid Peroxides (LPO)

The LPO level in cells was assessed using a lipid peroxidation MDA assay kit (Beyotime Biotechnology, China, S0131S). The drug-treated cells were lysed to make cell lysate, which was reacted with thibabituric acid (TBA) and the absorbance at 532 nm was measured with Molecular Devices.

### 2.11. Reactive Oxygen Species (ROS) Assay

DCFH-DA (Beyotime Biotechnology, China, S0033S), a fluorescent probe, was used to detect intracellular ROS. DCFH-DA dilution of 10 *μ*mol/ml was added to the six-well plates and it was incubated in a CO_2_ incubator for 20 mins. Finally, the fluorescence intensity was measured under a fluorescent microscope.

### 2.12. Iron Assay

FerroOrange (Dojindo Laboratories, Japan, M489) is a novel fluorescent probe for fluorescence imaging of Fe^2+^ in living cells. Drug-treated cells were washed three times with HBSS, then 1 *μ*mol/l FerroOrange working solution was added to the six-well plates. Finally, the six-well plates were incubated in a CO_2_ incubator for 30 minutes for imaging by fluorescence microscopy.

### 2.13. Statistical Analysis

All statistical analyses and visualizations were conducted with R software (version 4.0.3) or GraphPad Prism 9. Pearson correlation analysis was used to determine the correlation between FRGs and ferroptosis-related lncRNAs. The paired Student's *t*-tests were used to estimate the statistical significance of molecular expression between CC samples and normal tissues. The Kruskal-Wallis and one-way ANOVAs were conducted to compare the differences between two or more groups. A *p* value less than 0.05 was considered statistically significant (^*∗*^ <0.05, ^*∗∗*^ <0.01, ^*∗∗∗*^ <0.001). The calculation of the drug synergy of erastin with cisplatin was analyzed by Calcusyn software (version 2.1).

## 3. Results

### 3.1. Variations of Ferroptosis-Related Genes and Ferroptosis-Related Pathways

**Figure 1:**
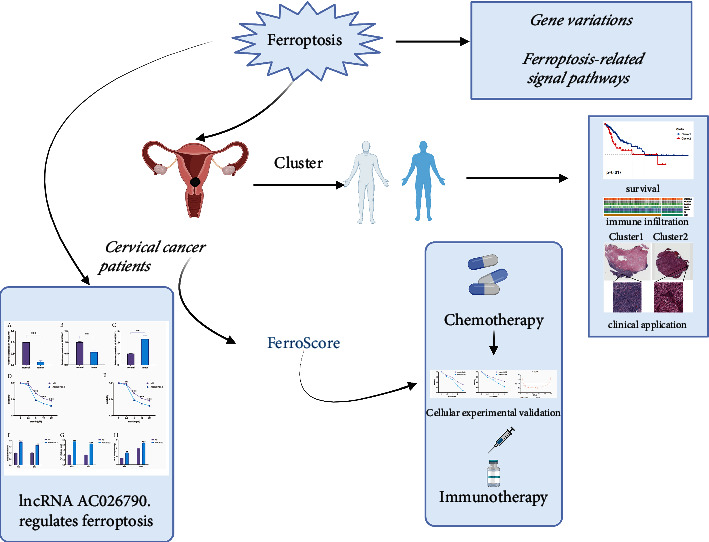
The overall flowchart of this article.

**Figure 2:**
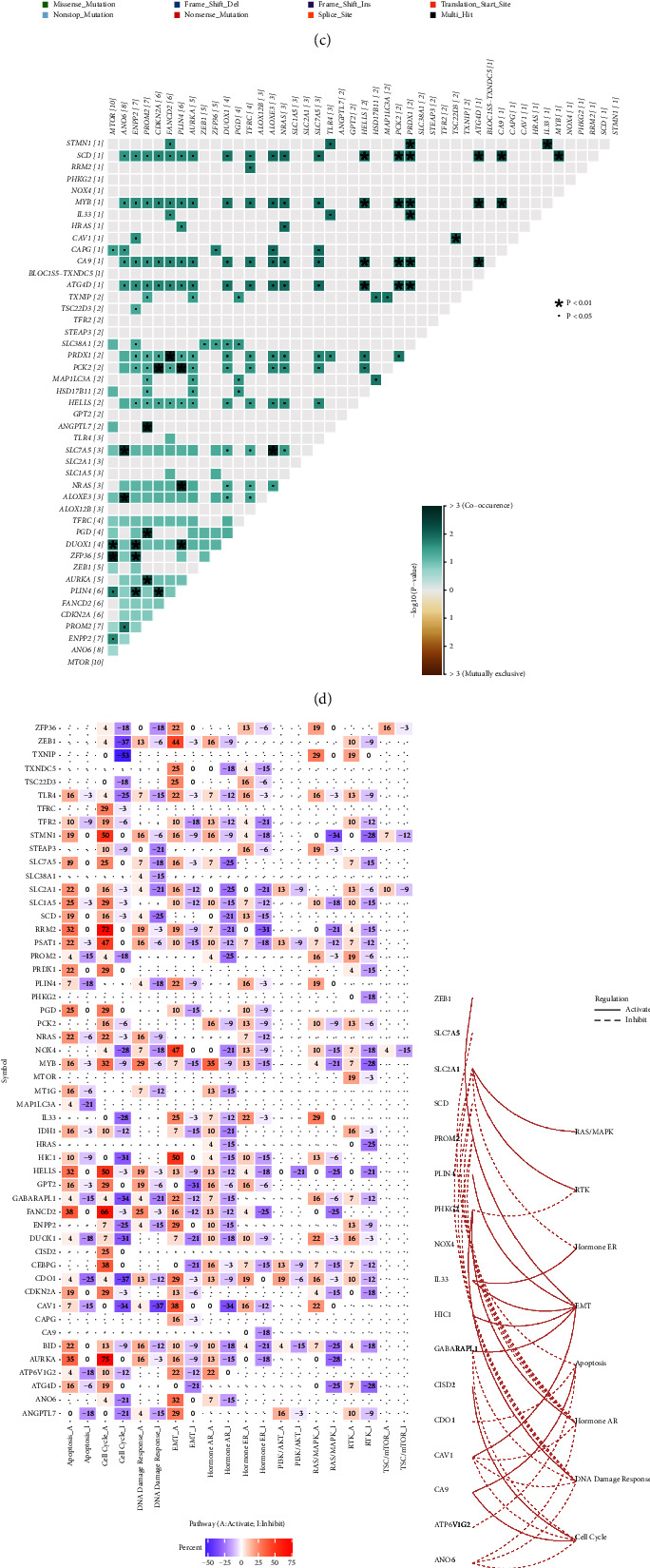
FRGs variations and associated pathways. (a, b). The correlation between CNV, methylation, and mRNA expression levels was represented, respectively. Red bubbles mean positive correlation, and blue means negative correlation. The darker the color means the larger the correlation coefficient. The size of the bubble means FDR. (c) Oncoplots of the somatic mutation displayed the frequency of 55 FRGs mutations in CC patients. (d) Mutations in 55 FRGs among CC patients. Asterisk shows *p* values (^*∗*^*p* < 0.01, *p* < 0.05) (e) The thermogram showed the correlation of the expression of 55 FRGs in the pan-cancer signaling pathway. Pathway_a (red) indicates the percentage of cancers in which the pathway is likely activated by a particular gene, inhibition in a similar way shown as pathway_i (blue). (f) The network showed the relationship between FRGs and pathways. The solid lines indicate activation and the dashed lines indicate inhibition. (g) GO analysis revealed that many biological processes related to metabolism and oxidative stress were enriched. (h) KEGG analysis showed that the ferroptosis pathway and immune checkpoint pathways were enriched.

The workflow of our study was shown in Figure 1. To identify differentially expressed FRGs (Supplementary Table S2), the cut-off of log FC > 1 and *p* < 0.05 were served as filter criteria for analysis with the “limma” package. CNV was positively correlated with the expression levels of mRNA in CC, and the analysis of CNV frequency changes showed that among the 55 FRGs, FANCD2 had the most frequency of CNV (Figure 2(a)). However, methylation level was inversely correlated with the most mRNAs expression levels in CC (Figure 2(b)). The frequency of mutated FRGs was 26.3% among the 289 samples, 76 of which had mutations. Among these 76 samples, MTOR had the highest mutation rate of 3%, mainly missense mutations (Figure 2(c)). There were significant comutations between DUOX1 and MTOR, CA9 and HELLS, ATG4D and PRDX1 (*p* < 0.01), etc (Figure 2(d)).

Almost all the FRGs in signaling pathways of pan-cancer, STMN1, RRM2, HELLS, FANCD2, and AURKA could significantly activate the cell cycle, but inhibit EMT, DNA damage response, hormones ER, RAS/MAPK, and RTK (Figure 2(e)). Most FRGs activated EMT signaling pathway but inhibited apoptosis, hormone AR, and DNA damage response signaling pathways in CC (Figure 2(f)). We enriched these 55 genes and the results of the KEGG enrichment analysis demonstrated that these genes were associated with ferroptosis, cancer-related signaling pathways, and immune checkpoints (Figures 2(g) and 2(h)).

### 3.2. Identification of Two Ferroptosis Clusters by NMF Algorithm

**Figure 3:**
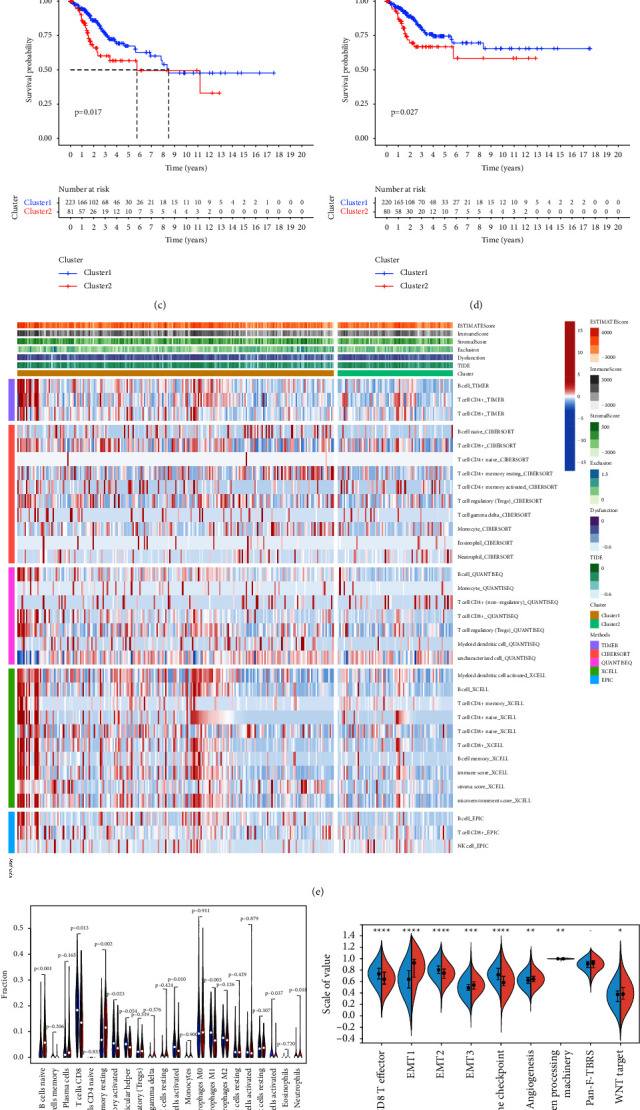
Relationship between two ferroptosis clusters and immune cell infiltration. (a) The relationship between cophenetic, dispersion, EVAR, residuals, RSS, silhouette coefficients, and sparseness with respect to a number of clusters. (b) The consensus map of NMF clustering results of patients with CC. Patients were classified into Cluster 1 and Cluster 2 based on the expression of 55 ferroptosis-related genes. (c, d). The Kaplan–Meier survival plot of OS (*p*=0.017) and DSS (*p*=0.027) in Cluster 1 and Cluster 2. (e) The heatmap showed the rate of immune cell infiltration in the two clusters and the immune score between the two clusters. (f) The boxplot compares the 22 immune cells between cluster 1 and cluster 2 in patients with CC. (g) The Wilcoxon test assesses the immune-related gene set scores between two clusters. (h) The pathological HE staining images of the two ferroptosis phenotypes.

The 55 differential FRGs were initially classified into molecular clusters by NMF consensus clustering, and the optimal values (*K*) of clusters were selected based on the NMF rank survey (*K* = 2) (Figures 3(a) and 3(b)). Therefore, CC patients were divided into two clusters, Cluster 1 and Cluster 2. Patients in Cluster 1 had better survival for both overall survival and disease-specific survival, while Cluster 2 had a bad prognosis (Figures 3(c) and 3(d)). The clinical data of the CC patients in the two Clusters were shown in Supplementary Table S3.

Immune infiltration assessment software such as Timer, CIBERSORT, QUANTISEQ, Xcell, and EPIC was used to calculate the level of immune cell infiltration in CC samples via RNA-Seq (Figure 3(e)). The results showed that the abundance of immune cells with antitumor activity, such as CD8+ T cells and CD4+ T cells was relatively higher in Cluster 1 than in Cluster 2. Moreover, there were obvious differences in the proportion of each immune cell between Cluster 1 and Cluster 2, such as B cells naive, T cells CD8, and NK cells activated, and the quantity of most immune cells in Cluster 1 was higher than that in Cluster 2 (Figure 3(f)). Immune-related gene set scores, which were calculated by the “GSVA” package, were compared between Cluster 1 and Cluster 2, and the scores of the CD8 T-effector and the immune checkpoints were higher in Cluster 1 (Figure 3(g)). As expected, more immune cells infiltration were observed in the tumor nests of Cluster 2 patients but less in the tumor tissues of Cluster 1 patients, as seen in the pathology slides (TCGA Diagnostic slides) (Figure 3(h)).

### 3.3. FerroScore Predicts Drug Sensitivity and Synergistic Effect between Erastin and Cisplatin

**Figure 4:**
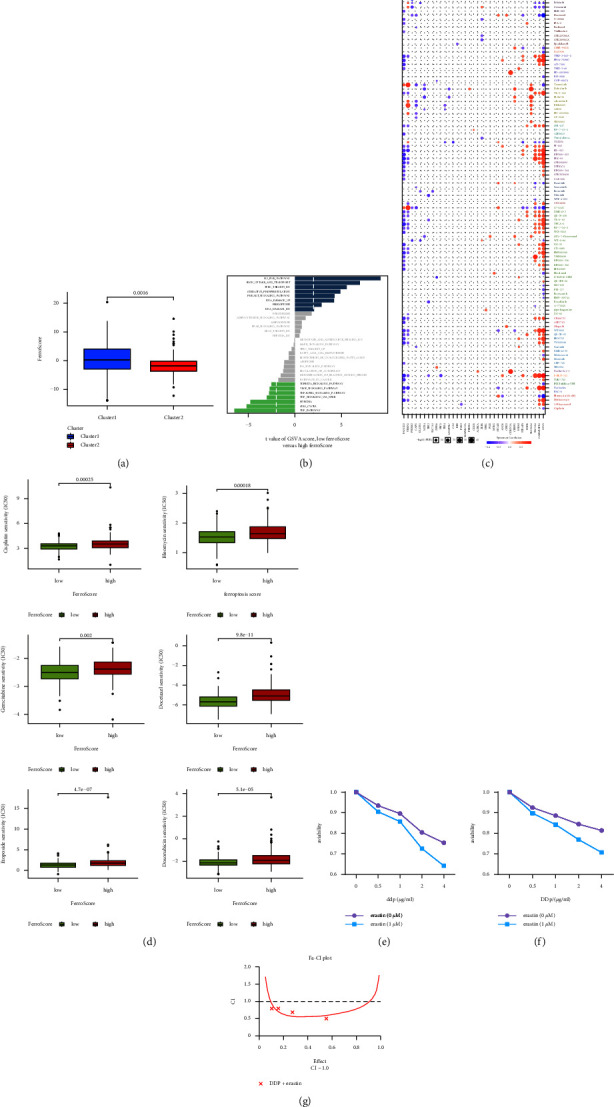
FerroScore predicted the response to chemotherapy and toxicity of erastin in combination with cisplatin on CC cells. (a) The Wilcoxon test showed the difference in PyroScore between the two clusters. (b) The bar graphs represented the distribution of t-values of the GSVA scores calculated for some pathways. (c) The bubble plot displayed the results of the correlation analysis of 55 FRGs for resistance to common clinical chemotherapy drugs in the GDSC database. (d) The Boxplot showed the difference in estimated IC50 values between the high- and low- FerroScore groups. (e, f). Different concentrations of cisplatin (0, 0.5, 1, 2 g/ml) in combination with erastin (0, 1 *μ*M) were used in Siha, Hela cells for 24 h after cell growth and proliferation inhibition. (g) Calcusyn software calculated the two-drug combination index of cisplatin and erastin, CI < 1 indicates synergy (0.8-0.9: low synergy, 0.6–0.8: moderate synergy, 0.4–0.6: high synergy, and 0.2–0.4: strong synergy).

A scoring system called FerroScore, the value of the sum of PCA1 and PCA2, was constructed using the PCA algorithm and calculated to evaluate the level of ferroptosis in each patient. According to the median score, CC patients were divided into two groups (high-FerroScore and low-FerroScore groups). The Sankey diagram presented the associations among clusters, FerroScore groups, and pathological types of CC (Supplementary Figures S1). A comparison of FerroScore in the two phenotypes indicated that FerroScore was higher in Cluster 1 than that in Cluster 2 (*p*=0.0016) (Figure 4(a)). We also analyzed the differences in some cancer-related pathways between high- and low-FerroScore groups, and the results showed that the low-FerrScore group was positively correlated with iron uptake and ferroptosis, while negatively correlated with TNF pathways (Figure 4(b)). Subsequently, the correlation of FRGs with drug resistance was analyzed using Spearman's correlation coefficient, according to drugs response data from the GDSC database. A correlation coefficient was positive, implying that the gene was highly expressed and resistant to the drug (Figure 4(c)). FANCD2 and STMN1 were synergistic for most drugs, while CAV1 and GABARAPL1 were antagonistic. CAV1 was strongly antagonistic to I-BET-762 and synergistic with Docetaxel. The differential drug resistance potential of the high- and low-FerroScore groups was further explored. We compared the estimated IC50 levels of several chemotherapeutic drugs in both groups. The six drugs had higher sensitivity in the low-FerroScore group, meaning that these six drugs were more effective in patients in the low-FerroScore group than in the high-FerroScore group (Figure 4(d)).

In vitro experiments, we further demonstrated that erastin-induced ferroptosis could increase the toxicity of cisplatin on Siha and Hela cell lines. It can be seen from the figure that the viability of the erastin + cisplatin group was significantly lower than that of the cisplatin group, which meant the erastin + cisplatin group had stronger toxicity to CC cells than the cisplatin group (Figures 4(e) and 4(f)). To further investigate whether there was a synergistic effect between erastin and cisplatin, we calculated the combination index (CI) of erastin and cisplatin for proliferation inhibition of CC cells using Calcusyn software (Figure 4(g)). The results showed that a CI value of less than 1 suggested a synergistic effect between the two drugs.

### 3.4. The Association between FerroScore and Immunotherapy in CC

**Figure 5:**
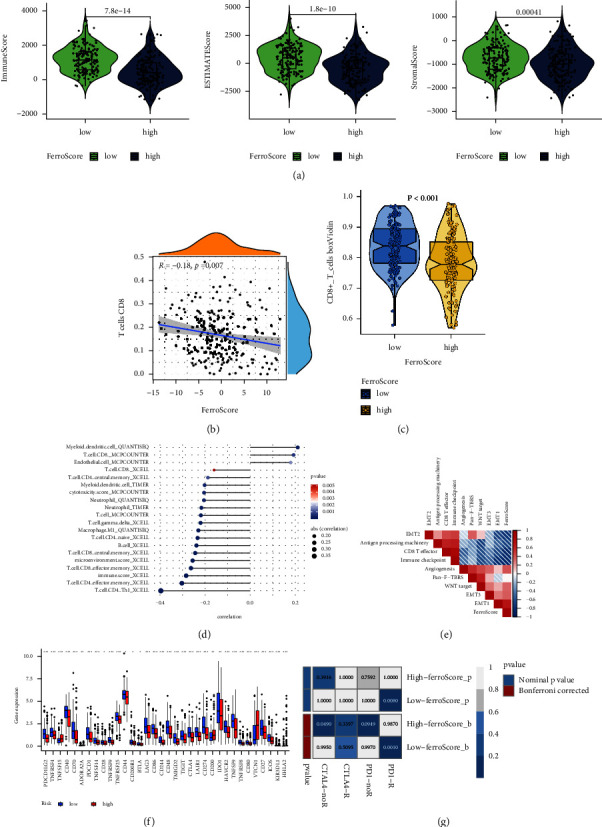
The relationship between TME, immunotherapy, and FerroScore. (a) The violin plot indicated the immune scores (stromal score, immune score, ESTIMATE score) between high- and low-FerroScore groups. (b) Relationship between the FerroScore and CD8+ cell infiltration. (c) Wilcoxon test of FerroScore variation of CD8+ cell. (d) The bubble plot revealed the correlation between FerroScore and the expression of immune cells. The color of the bubble means the *p* value; with darker blue indicating smaller *p* values. The larger the bubble, the stronger correlation. (e) The heat map showed the correlation between FerroScore and some immune-related scores. Red indicates a positive correlation, and blue is a negative correlation. The asterisk indicates *p* value. (f) The boxplots for the comparison of the immune checkpoint genes between the high- and low-FerroScore groups in the CC patients. (g) The submap algorithm predicted the likelihood of response to anti-PD1 and anti-CTLA4 immunotherapy in the high and low FerroScore groups. (^*∗*^*p* < 0.05, ^*∗∗*^*p* < 0.01, ^*∗∗∗*^*p* < 0.001).

We examined the association between the ESTIMATE score of the immune infiltration microenvironment and FerroScore. There were higher ESTIMATE scores, immune scores, and stromal scores in the low-FerroScore group than in the high-FerroScore group (Figure 5(a)). Several studies have demonstrated the enhanced role of CD8+ T cells in cancer immunotherapy. The correlation between FerroScore and CD8+ T cells in CC patients was investigated, and we found that patients with low-FerroScore had higher levels of infiltration of CD8+ T cells (Figures 5(b) and 5(c)). FerroScore was negatively correlated with the level of infiltration of the majority of immune cells but adversely correlated with the amount of myeloid dendritic cells (QUANTISEQ), T cell CD8 (MCPCOUNTER), and endothelial cell (MCPCOUNTER) (Figure 5(d)).

There was a significant difference between FerroScore and most immune-related scores, and FerroScore was negatively correlated with CD8 T effector and immune checkpoints (Figure 5(e)). Besides, the relative expression of immune checkpoints was evaluated in the high-FerroScore group and low-FerroScore group. Among them, most of the immune checkpoints were highly expressed in the low-FerroScore group, including PDCD1 (PD-1), CTLA4, and many other validated and effective immunotherapy targets (Figure 5(f)). A submap algorithm was used to predict the response of the high- and low-FerroScore groups to anti-PD1 and anti-CTLA4 immunotherapies (Figure 5(g)). The study proved that the low-FerroScore group may benefit more from anti-PD1 therapy. In summary, the above results strongly indicated that ferroptosis was associated with the CC immune microenvironment and response to anti-PD-1/L1 immunotherapy. In addition, FerroScore is a potential indicator of anti-PD-1/L1 immunotherapy.

### 3.5. AC026790.1 Overexpression Promoted Ferroptosis in CC Cells

**Figure 6:**
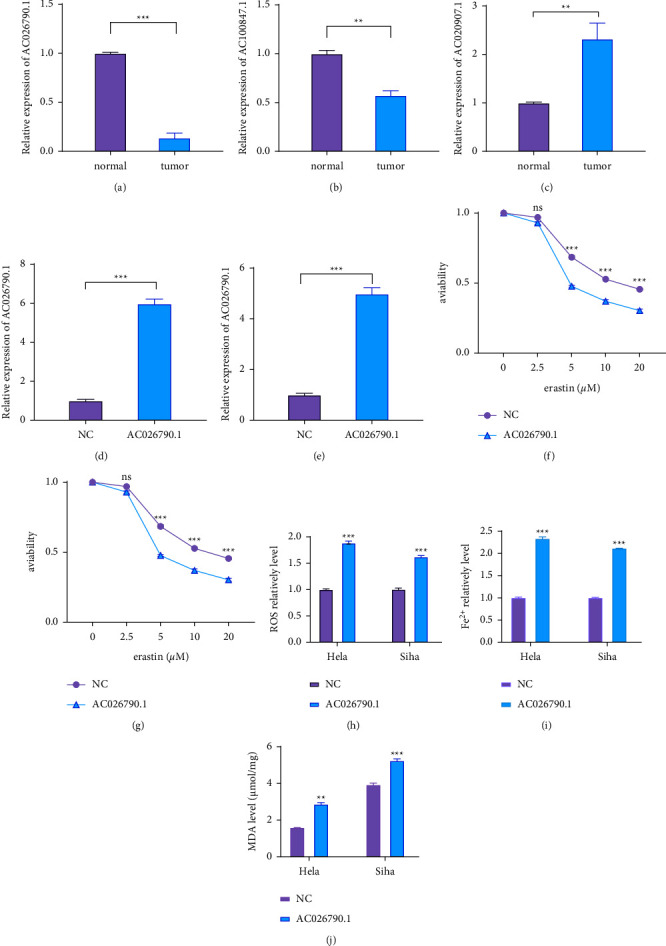
The regulation of erastin-induced ferroptosis by AC026790.1 (a∼c) The relative expression of AC026790.1, AC020907.1, and AC100847.1 in 20 pairs of CC tissues and their corresponding paracancer samples. (d, e) qRTPCR analysis was performed to detect the levels of AC026790.1 in Hela and Siha. (f, g) Overexpression of AC026790.1 transfected CC cells (Hela and Siha) were treated with different concentrations of erastin (0, 1, 2.5, 5, and 10 *μ*M) for 24 h, and cell viability was detected by CCK-8. (h–j) Overexpression of AC026790.1 in SiHa and HeLa cells detected differences in ROS, Fe^2+,^ and MDA between erastin-treated and NC groups. (^*∗∗*^*p* < 0.01, and ^*∗∗∗*^*p* < 0.001).

In total, 31 DE-lncRNAs were identified to be associated with ferroptosis. Especially, three ferroptosis-related lncRNAs (AC026790.1, AC100847.1, and AC020907.1) were related to prognosis by univariate cox analysis. And the expression of AC026790.1, AC100847.1, and AC020907.1 was validated in CC tissue samples. AC026790.1 and AC100847.1 were down-regulated in CC tissues compared to adjacent paracancerous tissues (Figures 6(a) and 6(b)), while AC020907.1 was upregulated (Figure 6(c)). Compared with normal cervical tissue, the difference in expression of AC026790.1 was more significant. Therefore, AC026790.1 was selected for further analysis.

To date, no research was available on AC026790.1 in CC and its potential function on erastin-induced ferroptosis. Then, we firstly used Hela and Siha cell lines to overexpress AC026790.1 and measured the efficiency of overexpression by qRT-PCR (Figures 6(d) and 6(e)). Subsequently, to understand the role of AC026790.1 in the regulation of ferroptosis, we investigated the viability of cells in the overexpression and negative control (NC) groups. AC026790.1 overexpression in HeLa and SiHa cells markedly facilitated ferroptosis compared with the NC group (Figures 6(f) and 6(g)). Next, three ferroptosis-related indicators were explored to further determine the regulatory role of AC026790.1 on erastin-induced ferroptosis. Iron is an essential micronutrient for the human body, but excess iron is associated with ROS production and cytotoxicity. As expected, it was observed that erastin-induced fluorescence of ROS and Fe^2+^ was increased significantly under fluorescence microscopy after overexpression of AC026790.1 (Figures 6(h) and 6(i). Impaired clearance or excessive production of LPO could lead to their accumulation, reaching lethal levels and triggering ferroptosis. The MDA kit was used to detect LPO, and MDA was significantly increased after overexpression of AC026790.1 after treatment with erastin in CC cells (Figure 6(j)). Therefore, it can be concluded that overexpression of AC026790.1 may contribute to erastin-induced ferroptosis.

## 4. Discussion

Ferroptosis is potentially a future silver bullet for a variety of cancers [24]. Induction of ferroptosis significantly inhibits tumor development and improves patient prognosis, even in cases of chemotherapy resistance, in which one of the primary mechanisms is apoptosis rather than ferroptosis [25, 26]. More and more evidence confirmed the critical role of ferroptosis in tumor immunity and enhanced tumor cell sensitivity to drugs, but the mechanism of FRGs in CC remains incompletely understood. In this study, we investigated the variation characteristics of FRGs in normal and tumor tissues in CC and concluded that the differences in FRGs expression may be associated with the regulation of genomic variations. Patients with CC were divided into two ferroptosis clusters, and we found significant differences in survival and immune cell infiltration between the two clusters.

Regarding the crucial role of ferroptosis in CC immune modulation and the individual cellular ferroptosis phenotype heterogeneity in CC, it was necessary to classify the expression of FRGs in CC patients. Therefore, we obtained FerroScore calculated by the PCA algorithm to evaluate FRGs in CC patients. There was an interaction between cells undergoing ferroptosis and remodeling of the immune microenvironment, as a recent study has shown [27]. Remarkably, we also revealed a correlation between FerroScore and the TME that might guide therapeutic treatments for patients in different groups. A number of studies have identified novel mechanisms of tumor suppression by CD8+ T cells through the induction of ferroptosis [28–30]. The expression of immune checkpoints such as CTLA 4 and PDCD-1 was significantly higher in the low-FerroScore than high-FerroScore group, implying that those patients may benefit more from checkpoint blockades [31], which were consistent with the prediction of the submap algorithm. Therefore, we hypothesize that the combination of ICIs with ferroptosis inducers has great potential and will contribute to the development of new combination therapeutic strategies and new immunotherapeutic agents.

Besides, the expression of three ferroptosis-related lncRNAs was also verified in CC samples [32–34]. Among them, lncRNA AC026790.1 was validated in several ferroptosis-related experiments to further elucidate its regulatory role in ferroptosis. The ferroptosis indicators such as MDA, Fe^2+^, and ROS were higher in the overexpression AC026790.1 group than in the NC group, indicating that overexpression of AC026790.1 promoted erastin-induced ferroptosis. The potential role of AC100847.1 and AC020907.1 on ferroptosis will continue to be explored in future experiments. Chemotherapy is currently one of the most effective methods of treating cancer. However, more and more patients are less effective due to apoptosis escape and drug tolerance [25, 35, 36]. However, numerous studies have demonstrated that the regulation of ferroptosis could influence the efficacy of tumor treatment and even reverse resistance to tumor treatment. Especially, three key pathways lipid metabolism pathway, GPX4-regulated pathway, and iron metabolism pathway mainly contribute to reverse chemoresistance [37]. The study showed that erastin-induced ferroptosis could synergistically enhance the toxicity of cisplatin on CC cells, which may provide a new therapeutic idea to overcome tumor drug resistance. Nevertheless, there are still some limitations in this article. Firstly, we only used 20 pairs of clinical samples to examine the relative expression of 3 lncRNAs, thus more samples would help make the results more reliable. Furthermore, the underlying regulatory mechanism of ferroptosis by AC026790.1 needs to be further illustrated.

## 5. Conclusion

In conclusion, our study showed that FRGs in CC were heterogeneous at the genetic variations, and revealed that two clusters had differences in immune cell infiltration in the TME. These results suggested that FerroScore improved the response of patients to chemotherapy even to chemotherapy resistance and immunotherapy. Our results showed that AC026790.1 could be a key molecule in regulating ferroptosis in CC and could be an effective target for CC treatment. LncRNA combined with FerroScore could improve patient prognosis and promote personalized treatment for CC patients.

## Data Availability

The data supporting the results of this study can be found in the article and can be consulted with the corresponding author.

## Supplementary Materials

Supplementary Materials
*Figure S1*. Sankey diagram showing the association between FerroScore and CC classification. *Table S1*. The primers for qRT-PCR. Table S2. The list of differentially FRGs. Table S3. The clinical characteristics of CC patients in Cluster 1 and Cluster 2.()
