# Supplementary material for: The Role of Ferroptosis-Related Molecules and Significance of Ferroptosis Score in Cervical Cancer
Source: J Oncol. 2022 Oct 30;2022:7835698. doi: 10.1155/2022/7835698 (PMC9637471; doi:10.1155/2022/7835698)
Supplement: Supplementary Materials — Figure S1. Sankey diagram showing the association between FerroScore and CC classification. Table S1. The primers for qRT-PCR. Table S2. The list of differentially FRGs. Table S3. The clinical characteristics of CC patients in Cluster 1 and Cluster 2.() [file 7835698.f1.zip › Table S1.docx]

Table S1: Primer sequences for qRT-PCR

| Gene ID | F | R |
| --- | --- | --- |
| AC026790.1 | ACTGTGATGCTTGATGGTCAACA | TCGTGGACAGTCAATGGCTG |
| AC020907.1 | ACCCAGGATGACTTCAGCGG | TTGTCTGGTGGGCACAGAGG |
| AC100847.1 | TGCACCAGGAGGTTTTATACATGAGG | CGCCTACTGGGTTCAAGTGATTCTC |
| GAPDH | ATGTTCGTCATGGGTGTGAAC | ATGGACTGTGGTCATGAGTCC |

F: forward primer; R: reverse primer
