# Supplementary material for: The Role of Ferroptosis-Related Molecules and Significance of Ferroptosis Score in Cervical Cancer
Source: J Oncol. 2022 Oct 30;2022:7835698. doi: 10.1155/2022/7835698 (PMC9637471; doi:10.1155/2022/7835698)
Supplement: Supplementary Materials — Figure S1. Sankey diagram showing the association between FerroScore and CC classification. Table S1. The primers for qRT-PCR. Table S2. The list of differentially FRGs. Table S3. The clinical characteristics of CC patients in Cluster 1 and Cluster 2.() [file 7835698.f1.zip › Table S2 (1).docx]

Table S2: The table of diferentially FRGs

| gene | conMean | treatMean | logFC | pValue | fdr |
| --- | --- | --- | --- | --- | --- |
| NOX4 | 1.376426 | 0.44288 | -1.63594 | 0.010611 | 0.042445 |
| DUOX1 | 1.027792 | 11.57248 | 3.493077 | 0.007684 | 0.041687 |
| PGD | 15.82474 | 107.4622 | 2.763576 | 0.003164 | 0.038074 |
| NRAS | 7.020174 | 19.321 | 1.460591 | 0.003896 | 0.038074 |
| HRAS | 14.97955 | 38.00431 | 1.343169 | 0.01143 | 0.042696 |
| TFRC | 9.115961 | 46.57165 | 2.352985 | 0.006973 | 0.04071 |
| TFR2 | 0.027294 | 1.640451 | 5.909376 | 0.003033 | 0.038074 |
| SLC38A1 | 6.417487 | 17.79254 | 1.471192 | 0.012532 | 0.044375 |
| SLC1A5 | 28.33844 | 96.52278 | 1.768109 | 0.008141 | 0.041687 |
| ALOX12B | 0.013614 | 2.626971 | 7.592165 | 0.007758 | 0.041687 |
| ALOXE3 | 0.032562 | 1.101222 | 5.079793 | 0.01348 | 0.046217 |
| PHKG2 | 2.493507 | 5.952757 | 1.255381 | 0.00344 | 0.038074 |
| ATG4D | 5.312774 | 13.91846 | 1.389463 | 0.009845 | 0.042445 |
| MAP1LC3A | 22.38366 | 9.612556 | -1.21945 | 0.01122 | 0.042696 |
| GABARAPL1 | 29.2483 | 11.00649 | -1.41 | 0.005079 | 0.038235 |
| BID | 3.347476 | 9.023209 | 1.430567 | 0.006448 | 0.039794 |
| ZEB1 | 17.28643 | 1.334132 | -3.69567 | 0.003164 | 0.038074 |
| CDKN2A | 0.398794 | 49.94334 | 6.968505 | 0.003299 | 0.038074 |
| CDO1 | 6.524798 | 0.33852 | -4.26862 | 0.003511 | 0.038074 |
| MYB | 0.123742 | 2.763494 | 4.481085 | 0.003369 | 0.038074 |
| ANO6 | 18.2872 | 7.924366 | -1.20647 | 0.010032 | 0.042445 |
| TLR4 | 3.747465 | 1.11799 | -1.74501 | 0.006706 | 0.040237 |
| IDH1 | 7.513336 | 24.15022 | 1.684511 | 0.005614 | 0.038235 |
| ANGPTL7 | 1.142189 | 0.04146 | -4.78395 | 0.005744 | 0.038235 |
| TSC22D3 | 74.9889 | 23.17879 | -1.69387 | 0.009129 | 0.042445 |
| PCK2 | 2.773701 | 11.67354 | 2.073358 | 0.003662 | 0.038074 |
| TXNIP | 556.9576 | 121.6473 | -2.19486 | 0.003896 | 0.038074 |
| GPT2 | 1.333697 | 8.76362 | 2.716096 | 0.003369 | 0.038074 |
| PSAT1 | 1.046236 | 27.05316 | 4.692517 | 0.003231 | 0.038074 |
| SLC7A5 | 5.5423 | 79.16847 | 3.836369 | 0.010032 | 0.042445 |
| ATP6V1G2 | 1.637217 | 0.222562 | -2.87896 | 0.008299 | 0.041687 |
| CEBPG | 8.53713 | 20.21648 | 1.243709 | 0.00423 | 0.038074 |
| BLOC1S5-TXNDC5 | 0.834614 | 0.096539 | -3.11192 | 0.008786 | 0.042174 |
| HSD17B11 | 44.55459 | 10.28083 | -2.11562 | 0.00423 | 0.038074 |
| IL33 | 31.50681 | 8.065911 | -1.96575 | 0.010222 | 0.042445 |
| STEAP3 | 3.693143 | 16.22782 | 2.135548 | 0.00725 | 0.041212 |
| SLC2A1 | 9.075772 | 146.575 | 4.013475 | 0.003978 | 0.038074 |
| PLIN4 | 12.19698 | 0.704548 | -4.11368 | 0.003369 | 0.038074 |
| HIC1 | 6.204416 | 0.745881 | -3.05628 | 0.003098 | 0.038074 |
| STMN1 | 18.58071 | 78.67942 | 2.082181 | 0.004145 | 0.038074 |
| RRM2 | 0.540237 | 34.93168 | 6.0148 | 0.002908 | 0.038074 |
| CAPG | 28.58334 | 118.4101 | 2.050546 | 0.00478 | 0.038235 |
| AURKA | 0.689412 | 15.50722 | 4.49143 | 0.002908 | 0.038074 |
| PRDX1 | 141.6567 | 294.3332 | 1.055051 | 0.01186 | 0.042696 |
| MT1G | 0.597083 | 17.90927 | 4.906632 | 0.005503 | 0.038235 |
| FANCD2 | 0.435973 | 4.350073 | 3.318728 | 0.002908 | 0.038074 |
| HELLS | 0.389836 | 5.23057 | 3.74603 | 0.00297 | 0.038074 |
| SCD | 5.118294 | 97.74308 | 4.25526 | 0.003033 | 0.038074 |
| MTOR | 3.345841 | 7.110571 | 1.087596 | 0.011643 | 0.042696 |
| ENPP2 | 35.63852 | 1.97441 | -4.17394 | 0.003164 | 0.038074 |
| CISD2 | 7.604858 | 16.88603 | 1.150837 | 0.005841 | 0.038235 |
| CA9 | 1.046794 | 44.69563 | 5.416085 | 0.01186 | 0.042696 |
| ZFP36 | 404.613 | 132.728 | -1.60807 | 0.014491 | 0.048155 |
| PROM2 | 4.059951 | 42.86244 | 3.40018 | 0.006448 | 0.039794 |
| CAV1 | 151.4043 | 29.61705 | -2.35391 | 0.004977 | 0.038235 |
