# Supplementary material for: The Role of Ferroptosis-Related Molecules and Significance of Ferroptosis Score in Cervical Cancer
Source: J Oncol. 2022 Oct 30;2022:7835698. doi: 10.1155/2022/7835698 (PMC9637471; doi:10.1155/2022/7835698)
Supplement: Supplementary Materials — Figure S1. Sankey diagram showing the association between FerroScore and CC classification. Table S1. The primers for qRT-PCR. Table S2. The list of differentially FRGs. Table S3. The clinical characteristics of CC patients in Cluster 1 and Cluster 2.() [file 7835698.f1.zip › Table S3 (1).docx]

Table S3: The clinical characteristics of CC patients in the Cluster 1 and Cluster 2.

| clinical characteristics | Cluster 1 | Cluster 2 | total |
| --- | --- | --- | --- |
| survival status | - | - | - |
| live | 178 | 56 | 304 |
| dead | 45 | 25 |  |
| age | - | - | - |
| <=60 | 181 | 64 | 304 |
| >60 | 42 | 17 |  |
| clinical stage | - | - | - |
| I | 128 | 34 | 304 |
| II | 47 | 22 |  |
| III | 32 | 13 |  |
| IV | 12 | 9 |  |
| unknow | 4 | 3 |  |
| T stage | - | - | - |
| T1 | 113 | 27 | 304 |
| T2 | 54 | 17 |  |
| T3 | 15 | 5 |  |
| T4 | 5 | 5 |  |
| TX | 11 | 7 |  |
| unknow | 25 | 20 |  |
| N stage | - | - | - |
| N0 | 107 | 26 | 304 |
| N1 | 46 | 14 |  |
| NX | 45 | 21 |  |
| unknow | 25 | 20 |  |
| M stage | - | - | - |
| M0 | 93 | 23 | 304 |
| M1 | 6 | 4 |  |
| MX | 95 | 33 |  |
| unknow | 29 | 21 |  |
