# Supplementary figures and images for: The Role of Ferroptosis-Related Molecules and Significance of Ferroptosis Score in Cervical Cancer
Source: J Oncol. 2022 Oct 30;2022:7835698. doi: 10.1155/2022/7835698 (PMC9637471; doi:10.1155/2022/7835698)

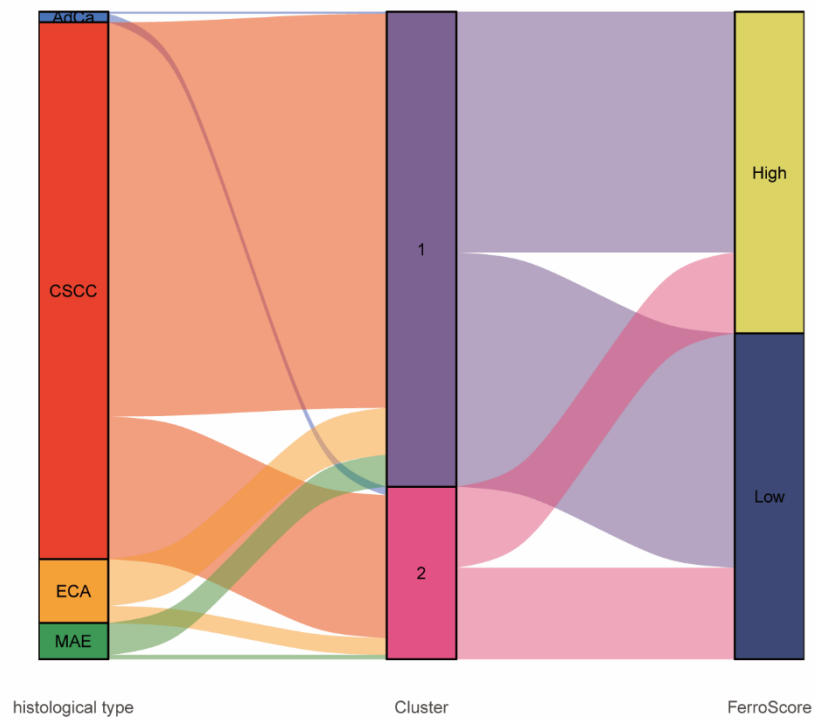

Figure S1: Sankey diagram showing the association between FerroScore and CC classification

Supplement: Supplementary Materials — Figure S1. Sankey diagram showing the association between FerroScore and CC classification. Table S1. The primers for qRT-PCR. Table S2. The list of differentially FRGs. Table S3. The clinical characteristics of CC patients in Cluster 1 and Cluster 2.() [file 7835698.f1.zip › Figure S1.pdf]
